# Adsorption of DDT and PCB by Nanomaterials from Residual Soil

**DOI:** 10.1371/journal.pone.0144071

**Published:** 2015-12-11

**Authors:** Mohd Raihan Taha, Shariat Mobasser

**Affiliations:** 1 Department of Civil and Structural Engineering, Universiti Kebangsaan Malaysia (UKM), Bangi, Selangor, Malaysia; 2 Institute for Environment and Development (LESTARI), Universiti Kebangsaan Malaysia (UKM), Bangi, Selangor, Malaysia; West Chester University of Pennsylvania, UNITED STATES

## Abstract

This paper presents the findings of a study on adsorption of dichlorodiphenythreechloroethen (DDT) and polychlorinated biphenyls (PCBs) on three nanomaterials including Multi walled Carbon Nanotube (MWNT), nano-clay and nano-alumina. DDT and PCBs are of significant concern due their high toxicity and long environmental half-lives. Experiments were conducted using batch adsorption procedures at different DDT and PCBs concentrations, from 10 to 60 mg/L. The amounts of MWNT, nano-clay and Nano-alumina used were 0.25%, 0.50%, 0.75%, 1%, 2% and 10%. The adsorption of PCBs solution onto the MWNT, nano-clay and nano-alumina was characterized by an initial rapid adsorption which eventually became constant within 22, 20, and 17 hours, respectively. The adsorption of DDT solution onto the MWNT, nano-clay and nano-alumina was also characterized by an initial rapid adsorption which gradually became constant within 22, 22 and 16 hours, respectively. Results of this study indicated that MWNT was a better adsorbent material compared to nano-clay and nano-alumina for both contaminants in this study. While at 10% of MWNT 88.9% and 77% of DDT and PCB were removed by MWNT, respectively. The effect of pH and temperature were also investigated.

## Introduction

In recent years, releases of different pollutants to the environment have drawn the attention of scientists due to their toxic effects [1]. Organochlorine (OC) compounds, such as dichlorodiphenyltrichloroethane and its metabolites (DDTs) and polychlorinated biphenyls (PCBs) are particularly given much consideration because of their capability to degrade environmental quality and cause ecological risks. These compounds are hardly soluble in water and resistant to biological, chemical and photolytic degradation [2]. Furthermore, Wang (2005) stated that organochlorine pesticides like DDT (C_14_H_9_Cl_5_) are bioaccumulative, and toxic, and are regularly seen in air, water bodies, sediment, and biota worldwide [3]. DDT is highly lipid soluble and is able to bioconcentrate and biomagnify in various tropic levels that lead to different toxic effects. The hydrophobic nature of DDT and its metabolites has led to significant accumulations in sediments. PCBs (C_12_H_10_−_x_Cl_x_) were utilized in several industrial fields such as electrical industry and hydraulic fluids, casting wax, carbonless carbon paper, compressors, heat transfer systems, pigments, fluoresecent light ballasts, etc.

Different systems can be considered for removing the environmental pollution. Adsorption is known as a popular method due to its accessibility and simplicity. Adsorption is increasingly employed for the elimination of both organic and inorganic contaminations found in water and soil. Features of adsorbent are significant in this process. The adsorbent must consist of influential traits in order to absorb the pollutants. Traditional remediation technologies have indicated confined efficacy in reduction of the concentration of contaminations in air, water, and soil. This particular study will engage a residual soil which is widely distributed in Malaysia.

According to Boehm (2006) nanomaterials can act more remarkably and influentially as filtration media in comparison with bigger particles with the same chemicals [4]. Nanoparticles have the ability to appear in a wide range of morphologies, from spheres to flakes, platelets, dendritic structures, tubes and rods. The unique structure and compounds of some nanoparticles establishes them as strong adsorbents, particularly for organic substances [5]. CNT (Carbon nanotube) have received much attention as very powerful adsorbents for a wide variety of organic compounds found in water. Examples include dioxin, polyaromatic hydrocarbon [6], dichlorodipheny trichloroethene, and its metabolites [7], PBDEs [8], chlorobenzenes and chlorophenols [9,10], trihalomethanes [11], bisphenol and nonylphenol [12], phthalate esters [12] dyes, pesticides i. e. thiamethoxam, imidacloprid and acetamiprid [7]. It was found that purification (removal of amorphous carbon) using of the CNT improved the adsorption of contaminant from environment [13]. As unique adsorbents, Multi Walled Carbon Nanotube (MWNT) were utilized to extract polybrominated diphenyl ethers from water and milk, chloropenols, phetalat esters and bisphenol. Also, MWNTs are able to extract a small diclofenac molecule from urine, three barbiturates and four benzodiazepine from pork. Moreover, MWNTs has potential to absorb the environmental pollutant due to their vast accessible particular surface area and hydrophobic surface [8, 14, 7, and 15].

Clay-based nanomaterials have become the focus of growing interest becaue of their nano size in their structural and functional properties. The nanometric scale of the expansive surface areas, anisotropic shape and reactive surface of clays with functional manner of organic polymer have been considered for developing organic and inorganic nanohybrid materials. The most commonly used clays as nano-adsorbents are montmorillonite/smectite and kaolinite group clays. Previous studies revealed that chemically modified clay minerals have become a new and encouraging class of sorbent materials [16]. As shao et al. (2009) observed, modified montmorillonite maintained high phenol adsorption capacity [17].

Nano-alumina, as an effective adsorbent for the adsorption of different analytes, was characterized by strong adsorption capacity, which could be attributed to its high surface area, porosity, degree of surface reactivity, mechanical strength and low temperature modifications. However, hydrophilic naked alumina nanoparticles do not represent perfect capacity of adsorption for organic substances [18]. It was found that clay could adsorb 87% of PCBs from soil and solution [19]. shao et al (2010) used montmorillonite for adsorption of phenol. In fact, the adsorption capacity of naked alumina nanoparticles for organic contaminants was not effective, because of the weak interaction between organic compound and the hydrophilic surface of alumina. To overcome this problem, chemical or physical modifications of the surface gamma-Al_2_O_3_ nanoparticles with certain functional groups containing some donor atoms were necessary [20]. According to Tchomgui-Kemaga et al. (2010) aluminum was an effective adsorbent for eliminating—fluoride from drinking water [21]. Furthermore, Bhatnagar et al. (2010) highlighted that nano-alumina could remove nitrates from solutions [22]. The pH and temperature are important parameters that have impact on remediation process by adsorption. In the various ranges of Temperatures and pH, adsorption efficiency will be changed. The proposed study will deal with PCB and DDT which contaminates soils and groundwater in Malaysia through unengineered landfills and the use of pesticides for agriculture. The study focused on removal of contaminant from residual soils in Malaysia. Therefore the condition of experiments is near the dominate temperature and pH range in Malaysia. The adsorption dynamic is popular method in remediation studies.

## Materials

Polychlorinated biphenyl was purchased from Sigma Adlrich, USA. The PCBs that was used in this study is 2-chlorobiphenyl. Its purity is 99.2%. The DDT was obtained from Sigma Aldrich, USA. The DDT type is 4, 4-DDT. Its purity is 98.2%. The DDT that was used in this study was 4, 4′-DDT. It has molecular weight of 354.49 g/mol. The PCB that was used in this study was C_12_H_9_Cl (2-chlorobiphenyl). It is 188.65 g/mol in molecular weight. The CNT used is a MWNT (Multi walled carbon nanotube) that was purchased from Arkema (France). The properties of MWNT used in this study are described in Table 1. Nano-clay was purchased from Southern Clay Products (USA). The nano-clay is Cloisite^®^ Na^+^ which is a natural montmorillonite. Nano-alumina (99.9%) was purchased as—ultra pure γ-Alumina (γ-Al_2_O_3_) powder from Inframat Advanced Material (USA). The trade name is Gamma Alumina.

**Table 1:** Some typical properties of the nanomaterials used in this study.

	MWNT	Nano-clay	Nano-alumina
**Average diameter**	10–20 nm	10–100 nm	10–30 nm
**Purity**	>90%	>90%	100%
**Form**	Powder	Powder	Powder
**Average thickness**	0.2–0.4nm	<2–6μm	20–50 nm
**Total surface area**	1200 m^2^/g	41.8039 m^2^/g	143.7037 m^2^/g
**Density**	50–400 kg/m^3^	28600 kg/ m^3^	3000 kg/ m^3^
**Max.tensile strain**	63GPa	4.656GPa	
**Colour**	Black	Off White	Off White

The centrifuge used was Kubota 5100. An Agilent 7890A Auto sampler gas chromatograph model 7890A equipped with 63Ni electron capture detector (ECD) was used to detect PCB and DDT residues in the soil solution samples. All data were acquired and analyzed using Agilent CHEMSTATION^®^ software (Agilent Corporation, MA, and USA). The limits of detection (LOD) for DDT and PCB were 0.05 and 0.09 μg/mL, respectively.

### Adsorption Analysis

The DDT and PCB solutions with different concentrations were prepared in triplicates. The concentrations of DDT and PCB used in this study were 10, 20, 30, 40, 50, 60 ppm. Also the various amounts of MWNT, nano-clay and nano-alumina, used in the experiments were 0.25%, 0.50%, 0.75%, 1%, 2% and 10% g of soil. The DDT solutions were added to the flask containing 12g of soil and various amounts of selected nanomaterials. Each test experiment was conducted in 2 phases- one phase with soil and the other—without soil. Adsorption amounts were measured from the difference between the results of experiments on samples that included soil and those without soil. The mixture was shaken until equilibrium was reached using a reciprocating shaker at 150 rpm. The adsorption equilibrium time was determined from the above equilibrium time. This was followed by centrifugation at 3000 rpm for 20 minutes thus the separation between liquid phase and solid phase was obvious. A blank sample was also included. The concentrations of DDT and PCB in the clear supernatant were determined by GC. The test tubes were taken out of the reciprocating shaker bath at every interval time and centrifuged at 3000 rpm for 10 minutes, to separate a top supernatant layer from a bottom layer of soil. The amount of DDT and PCB adsorbed on to the MWNT, nano-clay and nano-alumina were calculated from the difference between the initial concentration and the amount remaining in the supernatant. A set of controls (untreated soil) and blanks was included in all the experiments. The amount of adsorption at equilibrium, Q_e_ (mg/kg), was calculated using the formula below:

$$Q_{e}=\left(C_{0}-C_{e}\right)\frac{V}{W}$$ (1)

Where C_o_ and C_e_ (mg/L) represent the liquid phase concentrations of DDT at the initial stage and at equilibrium, respectively. *V* is volume of solution and *W* is the mass of soil (12g). Percentage of adsorption also was calculated using the formula below:

$$R\%=\left(C_{0}-C_{e}\right)\times \frac{100}{C_{e}}$$ (2)

The pH of the solution was adjusted (using diluted NH_3_ and CH_3_COOH) to 2, 3, 4 and 9, 10 for DDT and PCB, respectively. The test tubes and their contents were shaken using a controlled environment incubator shaker (150 rpm) at a constant temperature of 28°C. To investigate the effect of temperature on adsorption, experiments were carried out at 28°C, 30°C, 32°C and 34°C of the shaking equilibrium time. The supernatants were centrifuged for 20 minutes at 3500 rpm. Subsequently, 1 ml of the supernatant extract was filtered through a 0.45 μm nylon syringe filter (Millipore^®^, USA), and injected into the GC-ECD. A similar procedure was also carried out for both DDT and PCB adsorptions with the soil samples. Three untreated controls (without soil and nanoparticles) for both toxics were also maintained simultaneously [23].

### Characterization

The surface area of the nanomaterials was measured using the Brunauer-Emmett- Teller (BET) method (N_2_ adsorption) with a Gemini apparatus (Micromeritics 2010 Instrument Corporation, USA) in UKM, faculty of science, department of physics physics Lab. An isotherm was obtained by measuring the amount of gas adsorbed across a wide range of relative pressures at a constant temperature (N_2_, 77K). Conversely desorption isotherms were achieved by measuring gas removed as pressure is reduced. This technique encompasses external area and pore area evaluations to determine the total SSA in m^2^/g yielding, important information in studding the effect of surface porosity and particle. BET showed specific surface area.

The images of nanomaterials were prepared using Field Emission Scanning Microscopy (FESEM). After prepared them in ultra-sonic bath. The change in the soil agglomeration and formation of new materials can be observed by this analysis. FESEM was conducted to disclose the morphology of nanomaterials.

The physicochemical properties of soil samples are enumerated in Table 2. Some of the soil properties that were investigated in this study were pH, CEC (cation exchange capacity) and water content. All soil data are expressed on a dry weight basis. The bulk density of the soils was also recorded.

**Table 2:** Some soil properties.

Soil Properties	Value
**Organic Content (%)**	1.67
**pH**	4.89
**Cation Exchange Capacity (CEC)-(meq/100 g)**	8.9
**Clay fraction (%)**	26.9
**Silt fraction (%)**	20.5
**Sand fraction (%)**	52.51
**Liquid Limit (LL) (%)**	36.96
**Plastic Limit (PL) (%)**	19.80
**Plasticity Index**	16.96
**Optimum Water content (%)**	14.29
**Chemical Composition**	Concentration (%)
**SiO**_**2**_ **(%)**	62.7
**Al**_**2**_ **O**_**3**_ **(%)**	29.45
**Fe**_**2**_ **O**_**3**_ **(%)**	5.7
**CaO (%)**	0.03
**Na**_**2**_ **O (%)**	-
**MgO (%)**	0.58
**K**_**2**_ **O (%)**	0.76
**TiO**_**2**_ **(%)**	1.17
**Other**	0.05
**Unified Soil Classification System (USCS)**	SC
**Specific Gravity**	2.6

## Results and Discussions

### Particle Size

**Fig 1:**
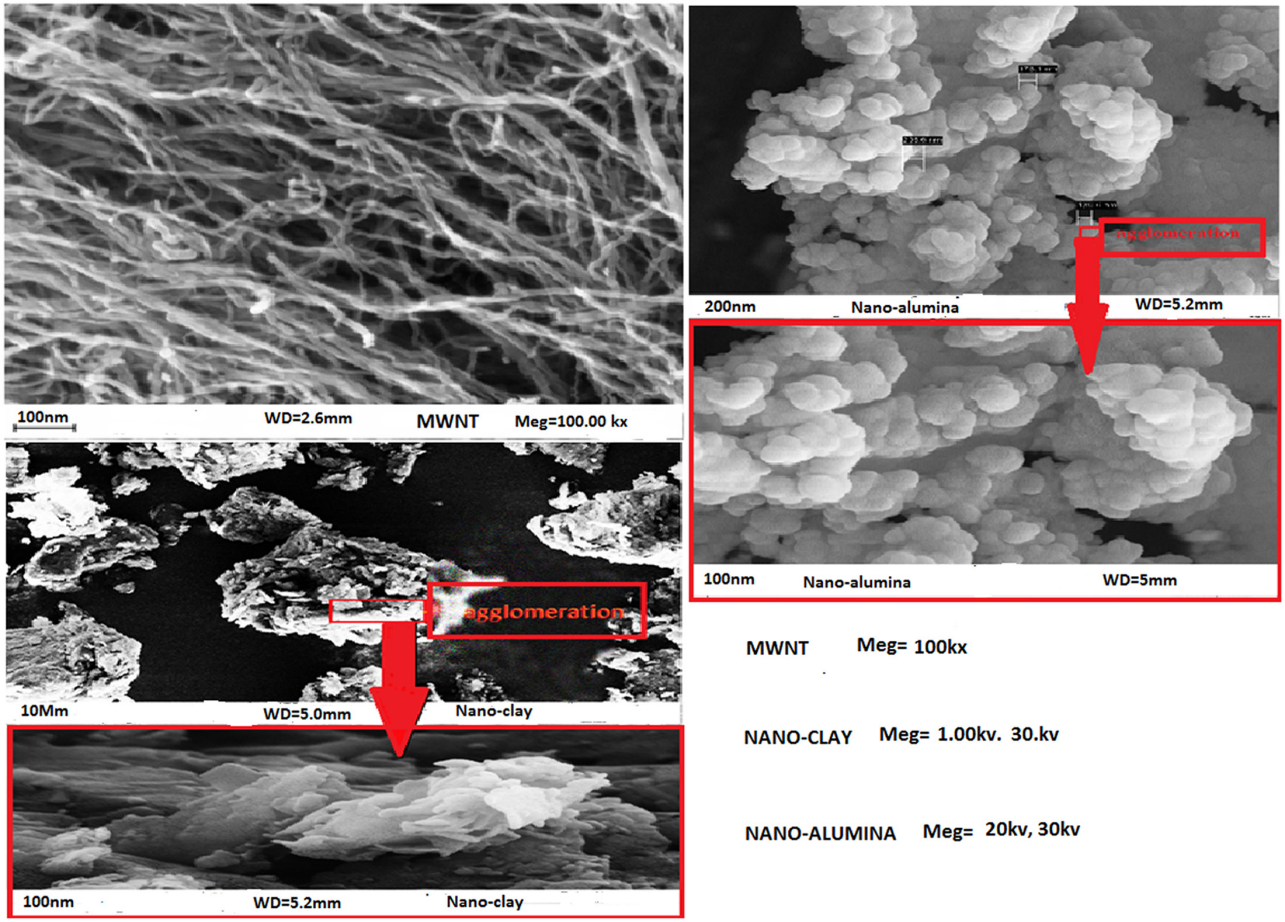
Fesem images of MWNT(a), nano-clay(b), nano-alumina (c).

In this study, FESEM was used to prepare the image of the nanomaterials. As shown in Fig 1a the multi-walled carbon nanotubes have uniform lengths—and diameters. Their lengths were about 300–400 nm and their diameters ranged from 10 nm to -20 nm. The large tubes were in fact agglomeration of much smaller tubes. Fig 1b and 1c show nano-clay and nano-alumina have high agglomerations and net uniform distributions. This agglomeration is shown clearly in the bottom image. The nano-alumina particles are shown to agglomerate together quite significantly. This value could be possibly lower than the actual because of particle agglomeration [24].

**Fig 2:**
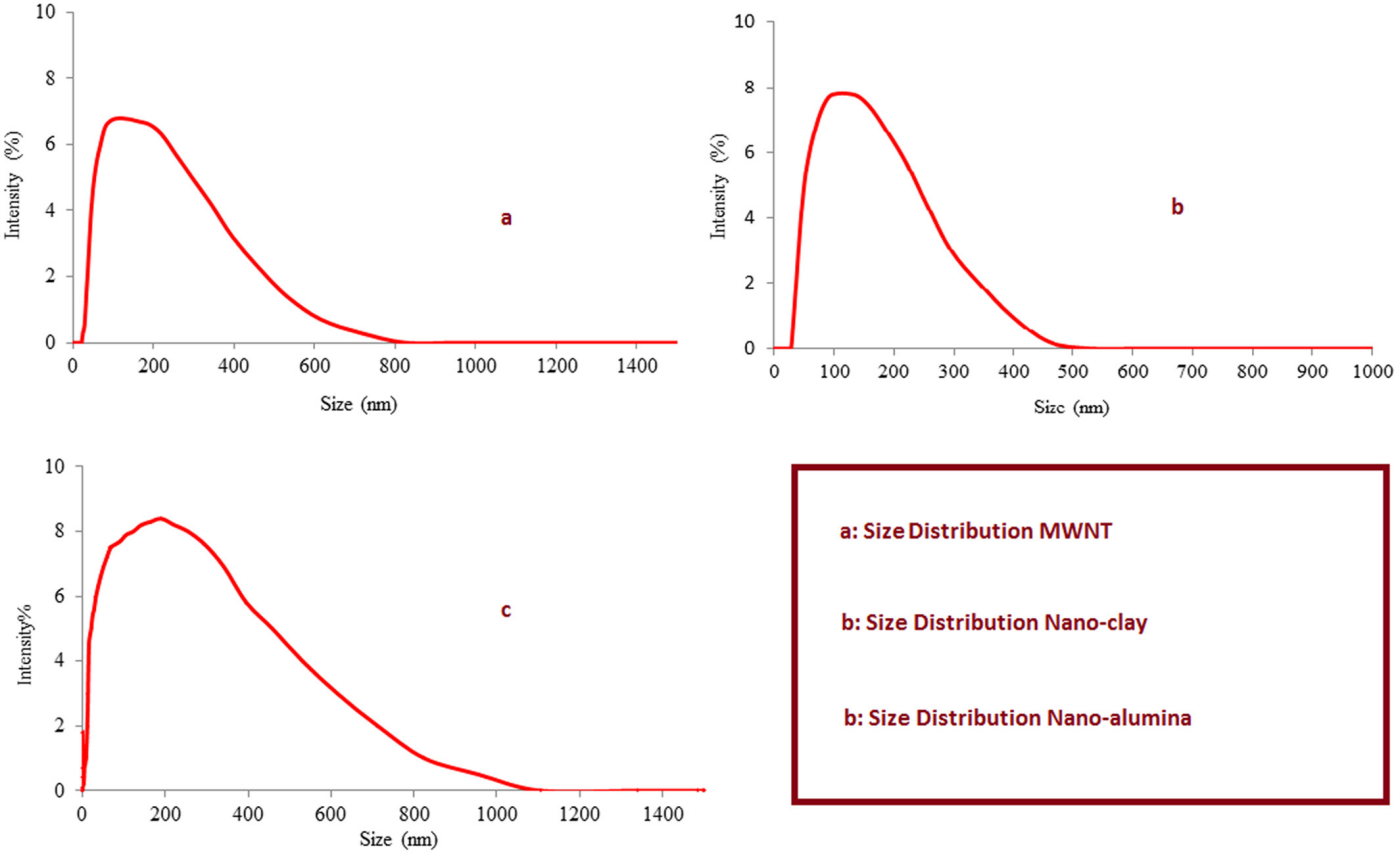
Size distributions of MWNT (a), nano-clay (b), nano-alumina (c).

The size distributions of the three nanomaterials used in this study were measured by zeta sizer instrument. As can be seen from the Fig 2, the highest intensity of size of MWNT, nano-clay, nano-alumina ranged from 10 nm to 400 nm, 10 nm to 400nm and 10 nm to 600 nm, respectively. It showed that the distribution of nano-alumina particles were wider and included both small and big size particles.

### Effect of contact time

**Fig 3:**
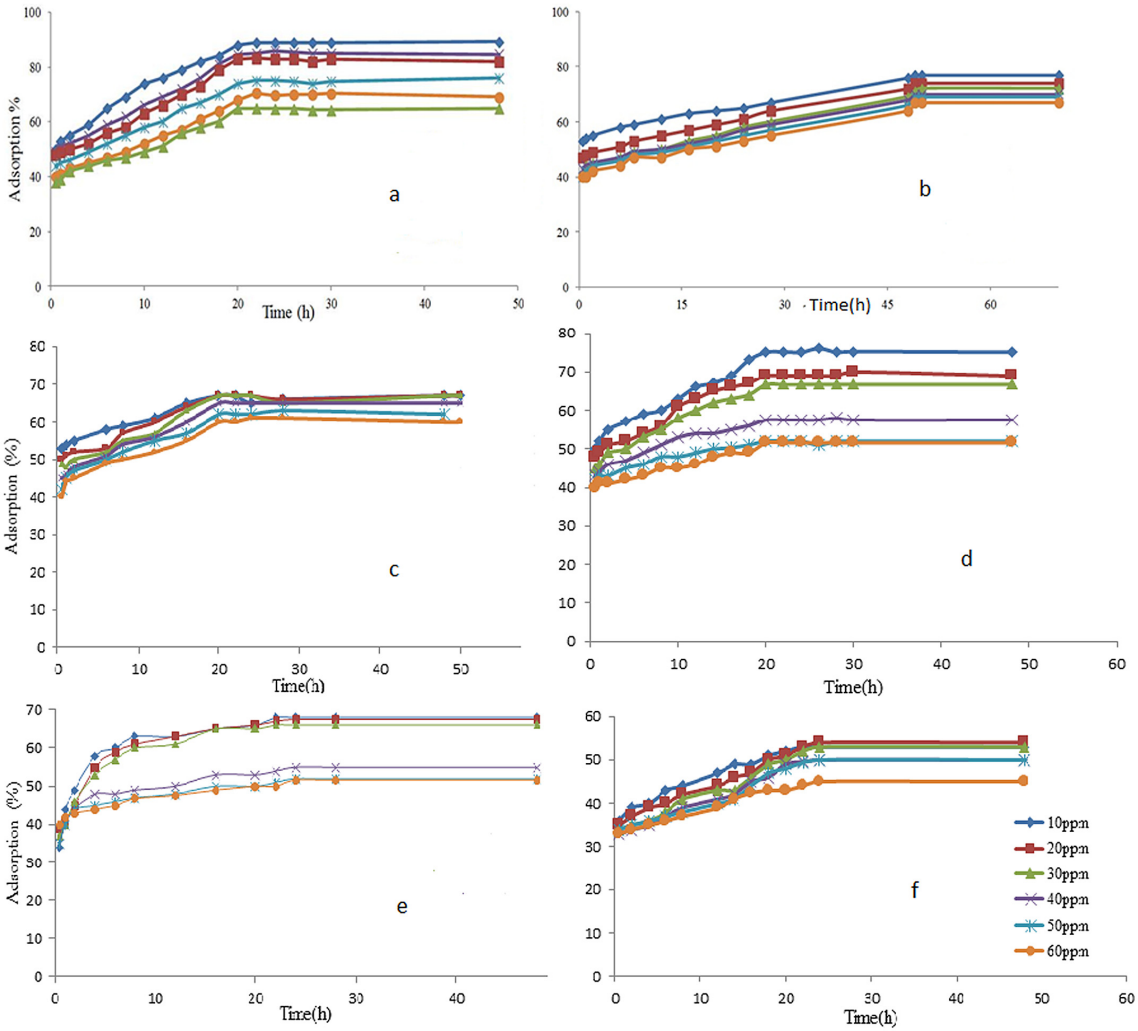
Effect of contact time on the amount of DDT (a) and PCB (b) adsorbed on MWNT, nano-clay and nano-alumina.

In order to determine the equilibrium (shaking) time for adsorption, the effect of contact time on adsorption was studied at room temperature. For initial concentrations of DDT and PCB in contact with MWNT, nano-clay and nano-alumina in this study, the results are shown in Fig 3. All concentrations of DDT and PCB in contact with various amount of nano materials (0.25% to 10%) were examined to find the effect of contact time. However at 10% of nanomaterials results were most clearly. It was found that the percentage of DDT adsorption on MWNT increased with time and slowly reaching equilibrium after about 22 h (hours). Thus, the 22 h period was established as the time in which the mixtures (soil-DDT-MWNT) were shaken for analysis of DDT adsorption with MWNT (Fig 3a). The initial pH values of (DDT+ soil) and (PCB+ soil) solutions recorded were between 4.5 and 4.9, respectively. The contact time for adsorption of PCB in this study was 49 h. Shao et al. (2010) reported that the equilibrium time for removal PCB occurred mainly in the first contact time of 24 h, and 45 h was enough to achieve the adsorption equilibrium [17]. Nollet et al. (2003) reported that the adsorption of 2, 3, 4-trichlorobiphenyl and 2, 2΄, 3, 3΄, 4, 5, 6- heptachlorobiphenyl on the fly ash reached equilibrium after contact time of 50h [25]. Moreover, 80–90% removal of 2, 3, and 4- trichlorobiphenyl occurred at that contact time. For all experiments conducted in this study, the rate of adsorption of PCB was slower than rate of DDT adsorption on MWNT. Fig 3 illustrates the effects of contact time on DDT and PCB adsorption on nano-clay. The percentage of adsorption of DDT and PCB increased with time and gradually reaching equilibrium after 22 h and 20 h, respectively. The results of of DDT and PCB in contact with nano-alumina are shown in Fig 3. It was found that the percentage of adsorption increased with time and slowly reaching equilibrium after about 16 hours. Thus, the 16 h period was established as the time in which the mixtures (soil-DDT-nano-alumina) were shaken for analysis. The results for the PCB series are shown in Fig 3b. As can be seen from the Fig 3, the percentage of adsorption increased with time and slowly reaching equilibrium after about 17 h.

### Adsorption percentage of contaminants by nanomaterials

**Fig 4:**
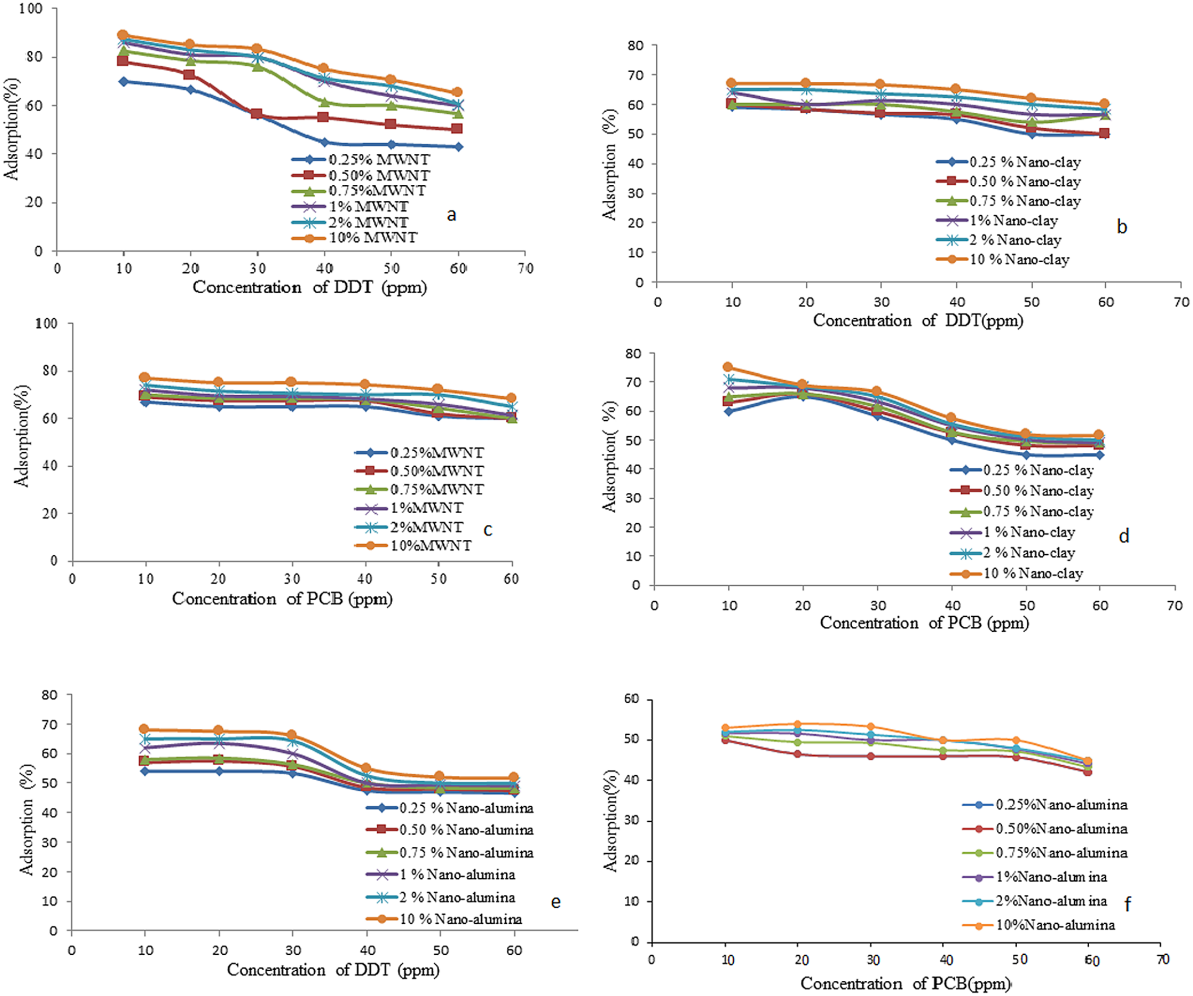
Adsorption of DDT and PCB in soil solution by various amounts of MWNT (a, b), nano-clay (c, d) and nano-alumina (e, f).

The results of the adsorption test conducted in this study are shown in Fig 4. The initial concentrations of DDT and PCB were between 10 ppm and 60 ppm. Experiments were conducted at room temperature and pH = 4.9. In cleaning-up of DDT and PCB from soil and soil solutions, the amount of adsorbent used in removal process is very important for economic reasons. Thereby, the effect of adsorbent dosage on adsorption of PCBs was first carried out.

Fig 4a illustrates that adsorption of DDT increased when the amounts of MWNT were raised. Maximum adsorptions were observed at the high levels of MWNT because of the maximum capacity of the adsorption sites. As shown in the figure, the maximum percentage of adsorption in this study was 88.9%. All the results showed that more than 50% of DDT was removed from the solution by 0.25% MWNT and more than 70% of DDT was eliminated from the solution by 10% MWNT. These indicated that MWNT had high adsorption capacity in immobilization DDT from large volumes of solution. The adsorption sites on the adsorbent surfaces were mainly composed of external surfaces, groove areas, and interstitial and inner pores sites. External surfaces and groove areas were usually available for adsorption, while for interstitial and inner pores, it was uncertain whether the molecules of persistent organic pollutants (POPs) were too large to fit into the area. Pan et al. (2005) found that DDT was able to fit into the surface and inner channels of MWNT [26]. Figure illustrates that the adsorption of PCB increased when the amount of MWNT was raised. As in the DDT series, the maximum adsorptions were observed at the high levels of MWNT because of the maximum capacity of the adsorption sites. As shown in Fig 4 more than 70% removal of PCB was removed from soil solution to % MWNT in the contact time. Wodarg et al. (2004) reported that particulate organic carbon had higher adsorption capacity for lower chlorinated PCBs than for higher chlorinated PCBs [27]. Sun et al. (2008) reported that removal of low chlorinated PCBs were much higher than that of high chlorinated PCBs by using activated carbon [28]. Here in, it is reasonable to mention that the adsorption of the monochlorobiphenyl, PCB used in this study by MWNT is high. Fig 3 illustrates that the adsorption of DDT increased when amount of nano-clay is increased. As shown in the Fig 4, the maximum percentage of adsorption for the parameters obtained from this study was 67%. All the results showed that more than 50% of DDT was removed from the solution by 10% nano-clay and more than 60% of DDT was eliminated from the solution by 10% nano-clay. The results of the adsorption of PCB on nano-clay test conducted in this study are shown in Fig 4. As shown in Fig 3, the maximum percentage of adsorption for the parameters in this study was 77%. The first phase of adsorption study include investigation of adsorption contaminants on nanomaterials without soil. The results show that all three nanomaterials are able to adsorb more than 70% of contaminants.

Fig 4 illustrates that adsorption of DDT increase when amount of nano-alumina is increased. The maximum adsorptions were observed at 10% of nano-alumina. The maximum percentage of adsorption for DDT on nano-alumina in this study was 68%. All the results indicated that more than 50% of DDT was eliminated from the solution by the 0.25% nano-alumina and more than 68% of DDT was -removed from the solution by 10% nano-alumina. As shown in the Fig 4, the maximum percentage of adsorption of PCB on nano-alumina in this study was 54%.

### Effect of pH

**Fig 5:**
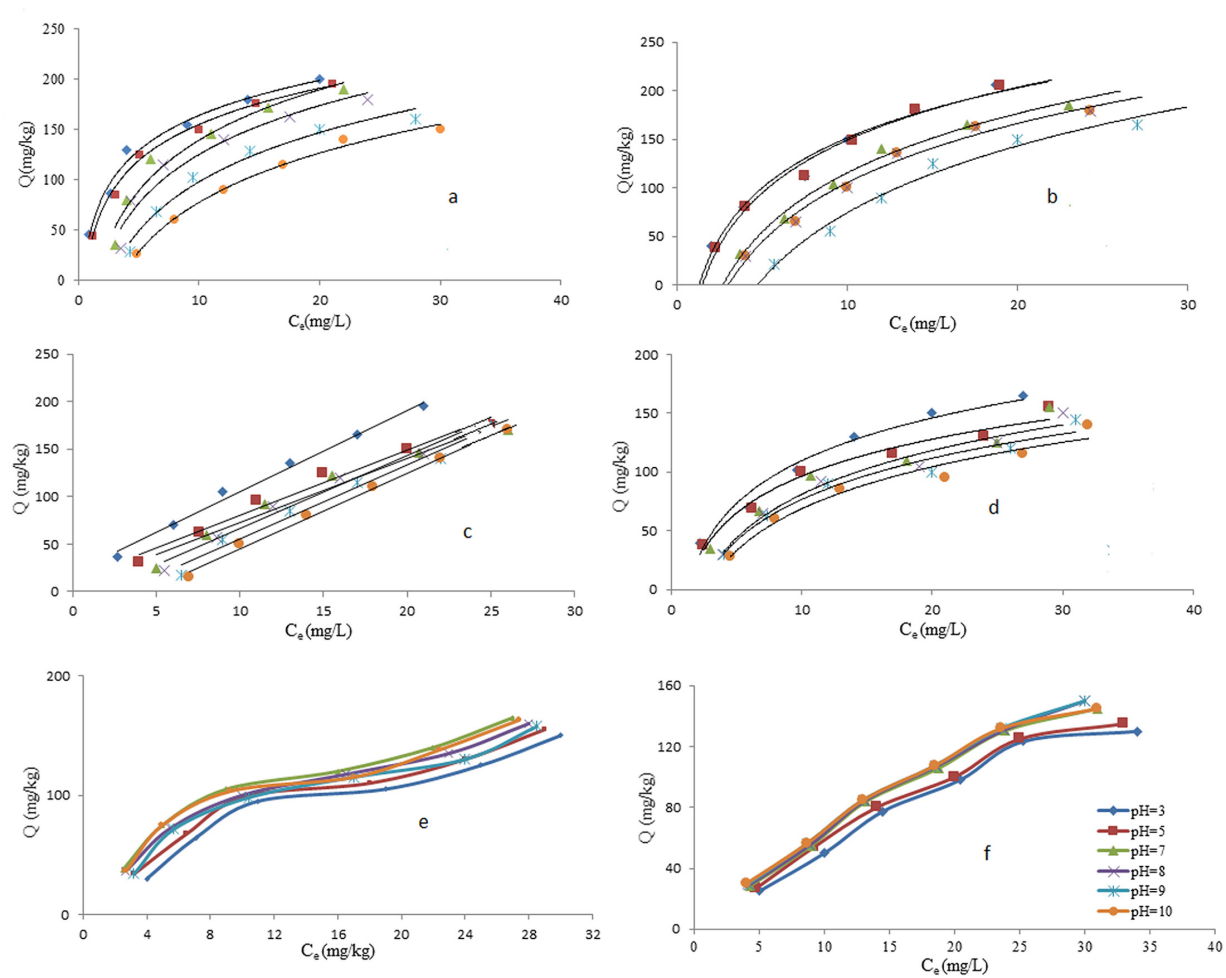
Effect of pH on the amount of adsorption of DDT and PCB on MWNT (a, b), nano-clay(c, d) and nano-alumina (e, f).

In this study, the effect of pH on the adsorption of DDT and PCB on to MWNT, nano-clay and nano-alumina surface was examined by varying the DDT and PCB-soil solution pH over the range of 3 to 10 at room temperature (28°C) in contact with 10% of each nanomaterial. The solution pH was one of the key factors that influenced the adsorption process on carbon materials by controlling the electrostatic interactions between the adsorbent and the adsorbate [29]. As illustrated in Fig 5, the amount of adsorption of DDT onto MWNT increased—with the decrease in the solution pH, i.e. the maximum uptake was noted at pH 3. Peng et al. (2003) explained this was possible due to the fact that more oxygen-containing groups on the MWNT surface were ionized at higher pH and thus they were able to adsorb more water [9]. The effects of pH on the removal of PCB from the contaminated soil to MWNT surface were studied by varying the solution pH over the range of 3 to 10 using the same concentration of PCB. As illustrated in Fig 5b, the amount of adsorption of PCB increased efficiently with the decrease of solution pH. The initial concentration was 10 mg/L at 28°C. The uptake of PCB by adsorbent in solution increased as the pH decreased from 10 to 3. Previous research had shown that the effects of solution pH on organic chemicals adsorption depended on the instability and electron—donor/acceptor ability of the organic chemicals [30]. The increase of solution pH of the ionizable solute led to increased ionization and hydrophilicity, and thus decreased the adsorption due to reduced hydrophobic interaction [15]. Increased pH would generally lead to higher ionization, solubility and hydrophilicity, and thus, decreased adsorption of natural organic compounds resorcinol [18], and herbicides, [31] on CNTs. Therefore, it was observed in this study that with increasing pH, the adsorption of PCB and DDT decreased directly. As illustrated in Fig 5c, the amount of adsorption of DDT on nano-clay increased efficiently with the decrease of solution pH. The initial concentration was 10 mg/L at 28°C. Maximum uptake (70%) was noted at pH 3. As illustrated in Fig 5d, the amount of adsorption of PCB increased with the decrease of solution pH. The initial concentration was 10 mg/L at 28°C. Maximum uptake (78%) was noted at pH 3. Results indicated that the adsorption of PCB decreased with increasing pH value. Analysis conducted in the study demonstrated that increasing pH value resulted in diminishing adsorption capacity. Wang et al. (2008) explained that increasing pH generally would lead—to increase ionization, solubility and hydrophilicity and thus would decrease the adsorption of organic compounds [23]. As illustrated in Fig 5e and 5f, the amount of adsorption of DDT and PCB increased with the increase of solution pH. The initial concentration was 10 mg/L at 28°C. A maximum uptake (70%) was noted at pH 7. The electron donor acceptor (EDA) interactions were suggested by Chen et al. (2008) as primary mechanisms for pH effects on adsorption. It explained that the effect of pH on organic chemical adsorption depended on the instability and electron-donor acceptor ability of adsorbent [30].

### Effect of Temperature

Temperature also is an important parameter that can influence the equilibrium and rate of environmental process. The effects of temperature on the equilibrium adsorption of DDT and PCB on the nanomaterials were studied at different temperatures, i.e. 28°C, 30°C, 32°C, and 34°C using a temperature-controlled water bath. The initial concentration of DDT was 10 mg/L and the MWNT dosage was 10% at pH = 4.9.

**Fig 6:**
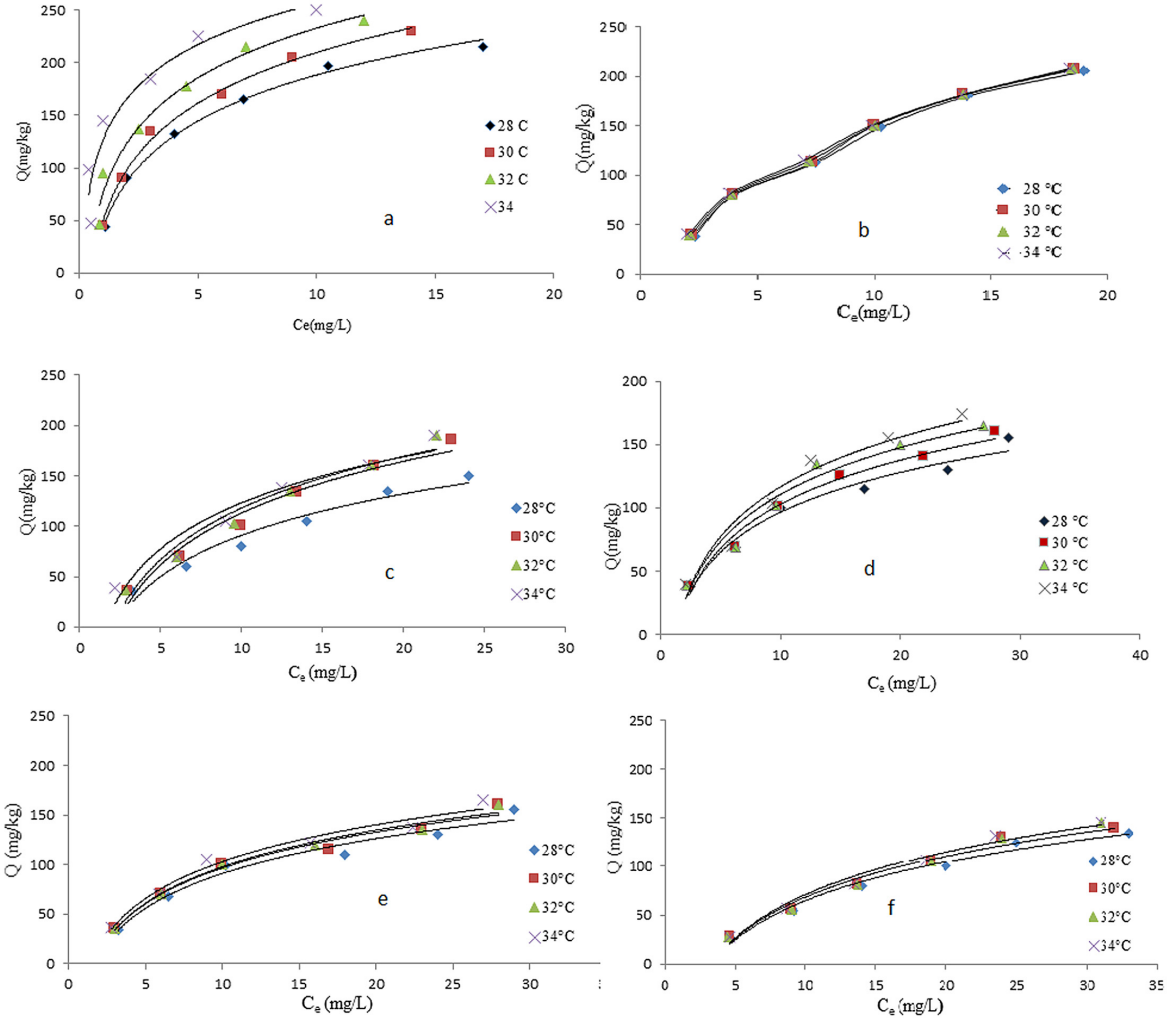
Effect of temperature on the amount of DDT and PCB on MWNT (a, b), nano-clay(c, d), nano-alumina (e, f).

As shown in Fig 6a, the amount of DDT adsorbed onto MWNT increased with increasing temperatures. When the temperature was increased from 28°C to 340C, the trend exhibited a gradual improvement of adsorption of DDT onto MWNT. The amount of adsorption increased from 44.45 mg/g to 47 mg/g by increasing temperature from 28°C to 34°C. It means that the percentage of adsorption is increased from 89% to 92%. Increasing the temperature is known to increase the rate of diffusion of adsorbate molecules across the internal pores of adsorbent particles and external boundary layer [32]. These phenomena could be explained by the fact that the kinetic energy of cation increased at higher temperatures, therefore, the contact between DDT and active site of adsorbent was sufficient, leading to increased adsorption efficiency [33]. The increase of adsorption with higher temperatures could also be due to the enlarged pore size of MWNT to some extent, which may also affect the carbon adsorption capacity. In addition, the increases in adsorption with temperature suggest that the adsorption process is endothermic, i.e. energy was absorbed [34]. Therefore, temperature had a positive effect on adsorption of DDT and PCB on MWNT. In this study, it was observed that due to the small range of temperature (the average temperatures in Malaysia); it has small effect on adsorption. As shown in Fig 6b, the PCB percentage of adsorption onto MWNT increases when the temperature is increased. When the temperature was increased from 28°C to 34°C, line exhibit a gradual improvement of percentage of adsorption onto MWNT. An increase in the amount of equilibrium adsorption of PCB with rise in temperature may be explained by the fact that the adsorbent sites were more active at the higher temperature [35]. The adsorption equilibrium depends on the temperature in two different ways. High temperature, generally, increases the rate of diffusion of the adsorbate molecules through the solution to the external and internal surface of the adsorbent and may change the equilibrium adsorption capacity of the adsorbent for the particular adsorbate [32]. The adsorption of PCB increases to 82% at 34°C. Similar trends have also been observed by other researchers for adsorption. In addition the increase of adsorption with temperature may enlarge the pore size of carbon nanotube to some extent, which may also affect carbon adsorption capacity [36]. Fig 6c illustrates the adsorption amount of DDT onto nano-clay as a function of temperature. A very small increase in the amount of equilibrium adsorption of DDT with rise in temperature may be explained by the fact that the adsorbent sites were more active at the higher temperature. It is found that the percentage adsorption of DDT increased from 67% to 69% when the temperature was raised from 28°C to 34°C, respectively. Fig 6d shows the adsorption amount of PCB onto nano-clay as a function of temperature. It was found that the percentage adsorption of PCB increased to 76% at 34°C. Schwarzenbach et al. (1993) explained that where sorption is an exothermic process, the equilibrium constant should decrease with increasing temperature. However, studies have also been reported where sorption increased with increasing temperature and where there was no effect of temperature [37, 5]. Results showed that the increasing of temperature had no significant effect on DDT and PCB adsorption. Fig 6e shows adsorption amount of DDT onto nano-alumina as a function of temperature. High temperature generally increase the rate of diffusion of the adsorbate molecules through the solution to external and internal surfaces of the adsorbent, and may change the equilibrium adsorption capacity of the adsorbent for the particular adsorbate [33]. Investigation of the results showed that the temperature has no significant effect on adsorption of DDT and PCB to nano-alumina.

### Absorption Capacity Analysis

In general, MWNT is a better adsorbent material compared to nano-clay and nano-alumina. Furthermore, MWNT adsorbed DDT better than PCB. In contrast, nano-clay exhibited greater adsorption potential for PCB than DDT. At 0.25% of MWNT, 70% and 68.3% of DDT and PCB were adsorbed, respectively, while at 10% of MWNT, 88.9% and 77% of DDT and PCB were removed by MWNT, respectively. This investigation confirmed that nano-clay was better adsorbent than nano-alumina for adsorbing PCB. Increasing the quantity of adsorbent resulted in an increase in the quantity of DDT and PCB that was removed from the solution. After MWNT, nano-clay was the better adsorbent for the contaminants tested. Anand (2010) explained the presence of high energy adsorption sites was typically ascribed as the reason for adsorption for different adsorbents [38]. With regards to MWNT, the high energy adsorption sites were related to the functional groups on the surface or the interstitial and groove regions between MWNT boundless. In this research, adsorption isotherms were studied to determine the sorption behavior and to estimate the adsorption capacity. Adsorption on the surface was governed by multiple intermolecular forces such as hydrophobic forces, electrostatic forces and hydrogen bonding [39]. Previous research has proved that π = π interactions could occur between molecules with C = C double bonds or benzene rings and MWNT surfaces. In this study, DDT and PCB both contain a benzene ring and therefore electrostatic forces such as π = π interactions would contribute to the adsorption of DDT and PCB. Besides the π = π interactions, DDT has five chlorines and PCB has a chlorine that are attached to its benzene ring. The π = π interactions has been used to interpret the adsorption of organic molecules with C = C double bond or benzene ring on the surface. This is because these organic molecules contain π electrons to interact with the π of the surface of adsorbent through the π = π electron coupling. The Cl is a typical electron acceptor and MWNT surface can serve as both an electron donor and electron acceptor. Therefore, an electron donor-acceptor (EDA) interaction is also likely between DDT and MWNT surfaces [40]. These increasingly stronger interactions are in agreement with the observed results that the adsorption capacity increased in an order of DDT > PCB for MWNT. These results indicated that MWNT will have important environmental impacts and its impact is dependent upon the nature of surface properties and aggregation structures. The changing sorption behavior of MWNT complicates the evaluation of fate and impact of MWNT in the environment. The adsorption capacity of PCB is smaller than DDT for both nano-clay and MWNT.

With increasing pH, the surface of MWNT and nano-clay becomes more negatively charged and the functional group on the adsorbate would be almost completely dissociated giving rise to repulsive effect and thus decrease adsorption. Below pH 6, the uptake of organic contaminants would slightly increase due to the better solubility of the compounds [30]. As in other acidic pesticides, there would be a reduction in adsorption as soil pH is increased, which is due to the lower adsorption potentials of the dissociated anionic forms compared with the undissociated molecular forms [41]. Peng et al. (2008) explained that the formation of water clusters on oxygen containing groups blocked the access of adsorbate to the adsorption sites of adsorbent, thereby resulting in less adsorption of adsorbate. The effect of temperature on adsorption was similar—in all three nanomaterials [9]. As the adsorption capacity increased at higher temperatures, it could be concluded that temperatures would lead—to higher chances for adsorbate to be adsorbed onto the adsorbents. Since the adsorption was endothermic, higher temperatures would result in high adsorptions [30]. However, in this study it is noted that the effect of temperature on adsorption was not significant because the temperature change is small.

## Conclusion

Comparisons among the three materials that were examined in this research showed that MWNT was the best adsorbent for DDT and PCB. It was found that DDT and PCB concentrations reduced significantly in the soil samples, when the amounts of nanomaterials were increased from 0.25% to 10%. It was found that the MWNT has high capacity to adsorb DDT from contaminated soil. The maximum percentage of adsorption of DDT by 10% of MWNT was about 89%. In addition, results show that the β parameter (maximum adsorption) increases from 149.25 to 250 mg/kg by increasing the amounts of 10% of MWNT. As an adsorbent for DDT, nano-clay and nano-alumina did not show excellent potential. Only 67% and 68% of DDT were adsorbed on nano-clay and nano-alumina, respectively. The maximum percentage of adsorption of PCB by MWNT was 77%. Results show that the β parameters is 909 mg/kg for PCB at 25% of MWNT. Results also indicated that nano-clay is an excellent adsorbent to remove PCB from soil and solution. Nano-clay adsorbed 75% of PCB at 10% nano-clay at 16 hours equilibrium time. Nano-alumina showed the lowest adsorption capacity amongst the nanomaterials (42% at 25% of nano-alumina). Comparison between three studied materials in this research shows MWNT is the best adsorbent for DDT and PCB. It was found that DDT and PCB concentrations reduced significantly in the soil sample, by increasing the amount of nanomaterials from 0.25% to 10%. The effectiveness of the treatment depends not only on the properties of adsorbent, but also on following environmental conditions and variables used for the adsorption process such as pH, temperature, contact time, and initial concentration. Results show that increasing pH has negative effects on adsorption of DDT to MWNT and nano-clay. However, pH has a positive effect on adsorption of DDT and PCB on nano-alumina. The effectiveness of the treatment depended not only on the properties of adsorbent, but also on environmental conditions and variables used for the adsorption process such as pH, temperature, contact time and initial concentration. Results illustrated that increasing of pH had negative effect on adsorption of DDT to MWNT and nano-clay. However, pH had positive effect on adsorption of DDT and PCB on nano-alumina. Results also showed that increasing the temperature from 28°C to 34°C (The temperature rang in Malaysia) had small positive effect (It is not significant effect) on adsorption of DDT and PCB by MWNT, nano-clay and nano-alumina. To analysis of result four adsorption isotherm were used. All isotherm have showed high R^2^ for adsorption DDT and PCB. The Langmuir adsorption isotherm showed the best fitting with data. Result showed that the nanomaterial used in this study are suitable materials to adsorb contaminants such as DDT and PCB.
